# N95 masks worn to protect against COVID-19 prevented tuberculosis exposure in healthcare workers

**DOI:** 10.1016/j.amsu.2022.103515

**Published:** 2022-03-28

**Authors:** Masaatsu Kuwahara, Chikako Takahashi, Sota Nishimura, Takashi Shinkai, Mitsuki Noma, Takuya Sunakawa, Misa Shimizu, Jun-Ichi Hirata

**Affiliations:** Department of Emergency and Critical Care Medicine, Hyogo College of Medicine, 1-1 Mukogawa-cho, Nishinomiya, Hyogo, 663-8501, Japan

**Keywords:** COVID-19 pandemic, N95, Tuberculosis, Prehospital medical care

## Abstract

Since the start of the COVID-19 pandemic, the healthcare workers in our institution have been equipped with N95 masks when performing aerosol-generating procedures, as these are associated with an increased risk of infection. We present a case in which using an N95 mask prevented tuberculosis (TB) exposure among healthcare workers administering prehospital care in rapid response vehicles. Even after the resolution of the COVID-19 pandemic in the future, wearing N95 masks among healthcare workers is recommended to protect against pathogens, including TB, when performing aerosol-producing procedures or prehospital activities for patients suspected of having respiratory diseases.

To the Editor,

We provide prehospital medical care for endogenous and exogenous emergencies with our rapid response vehicle service with one to two doctors and one nurse. The world is currently going through the coronavirus disease (COVID-19) pandemic. The pandemic has brought about changes in infection control in emergency rooms, including prehospital activities. Before the pandemic, wearing personal protective equipment was a standard precaution in prehospital settings. Surgical masks were used, but in some cases, no masks were worn. Since COVID-19 is spread via aerosols, healthcare workers have to wear the appropriate personal protective equipment, specifically N95 or other respirators (powered air-purifying respirators), when performing aerosol-generating procedures [[1], [2], [3], [4]]. Since the start of the COVID-19 pandemic, the healthcare workers in our institution have been equipped with N95 masks when performing aerosol-generating procedures, as these are associated with an increased risk of infection. We present a case in which using an N95 mask prevented tuberculosis (TB) exposure among healthcare workers administering prehospital care in rapid response vehicles.

A man in his 30s went into cardiac arrest at home, and medical care was immediately requested. Two doctors and one nurse were dispatched to administer prehospital care. Aerosol-generating procedures, such as cardiopulmonary resuscitation and tracheal intubation, were necessary. Therefore, the dispatched healthcare workers were equipped with N95 masks. However, the patient was not in cardiac arrest. He had developed respiratory failure and impaired consciousness. Since the patient had a respiratory problem, COVID-19 was suspected; thus, N95 masks were kept intact. However, miliary TB, pulmonary TB, and TB meningitis were diagnosed, and the patient tested negative for COVID-19. The healthcare workers had worn their N95 masks throughout the course of prehospital care delivery, i.e., until in-hospital treatment was administered. Therefore, there was no exposure to *Mycobacterium tuberculosis* among the healthcare workers.

Tracheal intubation and sputum induction promote droplet dispersion and increase the risk of TB infection [5].

Prior to the COVID-19 pandemic, the healthcare workers administering prehospital care to patients suspected of having a cardiac arrest were equipped with surgical masks. This level of protection is inadequate against *M. Tuberculosis* infections. Thus, the likelihood of exposure among healthcare workers involved before diagnosis increases. While N95 masks cost 30 times more than surgical masks, preventing TB exposure among healthcare workers significantly outweighs the cost disadvantage.

The TB prevalence in developed countries, including Europe and the United States, is less than 10 per 100,000 population. However, Japan reportedly had a medium prevalence of 13.3 per 100,000 population in 2017 and 16,789 reported cases. Our emergency department had six reported TB cases brought in 4 years before the COVID-19 pandemic. After the COVID-19 pandemic, we had only one case of tuberculosis (TB), and we wore N95 masks for protection. The lack of awareness and vigilance about TB among the general public and healthcare workers results in delayed detection and mass infection. Even after the resolution of the COVID-19 pandemic in the future, wearing N95 masks among healthcare workers is recommended to protect against pathogens, including TB, when performing aerosol-producing procedures or prehospital activities for patients suspected of having respiratory diseases.

## Ethical approval

No ethical approval was required.

## Please state any sources of funding for your research

No funding source.

## Author contributions

MK has prepared the paper. Other authors: Medical practice performed the data collection.

## Registration of research studies

This is a case report and has not been registered.

1. Name of the registry:
2. Unique Identifying number or registration ID:
3. Hyperlink to your specific registration (must be publicly accessible and will be checked):

## Guarantor

Masaatsu Kuwahara and Jun-ichi Hirata.

## Consent

Written informed consent was obtained from the patient for publication of this case report and accompanying images. A copy of the written consent is available for review by the Editor-in-Chief of this journal on request.

## Provenance and peer review

Not commissioned, externally peer reviewed.

## Declaration of competing interest

There are no conflicts of interest.
